# Changes in trends of visits and service utilization by mental health patients in the community: a twelve-year study in Israel

**DOI:** 10.1186/s13584-025-00735-y

**Published:** 2025-12-19

**Authors:** Marina Mor Shalom, Avner Kantor, Eyal Azuri, Daniella Tsulker Pirian, Jennifer Kertes, Beatriz Hemo, Tali Shmueli

**Affiliations:** 1 Health Division, Maccabi Healthcare Services, 27 Hamered Street, 6812509 Tel Aviv, Israel; 2 Ariel University, Tel Aviv, Israel

**Keywords:** Mental health, Primary care, Depression, Anxiety, Mental health reform, Covid-19

## Abstract

**Background:**

Mental health conditions, notably depression and anxiety, affect one in eight individuals globally, with 17.6% of Israelis reporting mood and anxiety disorders. Rising multimorbidity in primary care, with poorer health outcomes when combined with depression or anxiety, poses a challenge for primary care physicians (PCPs). This study aimed to track patient visits for depression and anxiety to PCPs over a period that included a national mental health reform, the COVID-19 pandemic, and the Iron Swords war, to characterize these patients and describe their health care service utilization.

**Methods:**

This observational study used data from Maccabi Healthcare Services, which serves over 2.7 million people in Israel. The study included patients aged eighteen and older who visited a PCP between 2013 and 2024. Patients were categorized into three groups for each year: depression/anxiety, severe mental illness (SMI), and a comparison group. ANOVA was used to compare mean PCP visit rates, and logistic regression to compare service utilization between the three groups.

**Results:**

Depression and anxiety patients were mainly female, older, of lower socioeconomic status, and had chronic illness. From 2013 to 2024, the number of patients increased, particularly during the COVID-19 pandemic and the Iron Swords war. Medical service utilization among these patients was more frequent than in the general population, averaging 14.2 primary care visits per year compared to 9.8, higher rates of emergency room and secondary care visits, hospitalizations, and polypharmacy.

**Conclusion:**

The study underscores the increasing impact of mental health conditions on primary care services in Israel between 2013–2014, along with the higher utilization rates of health services among anxiety and depression patients. The findings highlight the need for comprehensive care provided by primary care physicians (PCPs) who are proficient in both physical and mental health. Additionally, they stress the importance of enhancing access to community-based mental health interventions. Policy changes are recommended to improve the availability of mental health therapists and enhance PCP training programs to equip physicians better to treat patients with depression and anxiety.

**Supplementary Information:**

The online version contains supplementary material available at 10.1186/s13584-025-00735-y.

## Background

Approximately one in eight individuals globally is diagnosed with a mental health condition, with depression and anxiety being the most common disorders [1]. A survey conducted in Israel found that 17.6% of respondents reported a lifetime occurrence of mood and anxiety disorders [2]. The incidence of multimorbidity among patients is increasing in primary care, with the combination of depression or anxiety leading to poorer health outcomes [3–11]. Consequently, this patient group poses a growing challenge for primary healthcare workers [10, 12, 13].

Several RCTs have been conducted in people aged 18 years and older with depression and/or anxiety along with at least one other chronic illness (e.g., diabetes, cardiovascular disease, hypertension, and chronic pain) to assess the effectiveness of psychological or pharmacological interventions within the primary care community settings [14–20]. These studies indicated that such care may help improve both mental and physical health disorders [12, 14, 21].

In the past decade, three major events may have influenced depression and anxiety diagnosis rates by primary care physicians (PCPs) in Israel. Firstly, on July 1 st, 2015, the Israeli Ministry of Health implemented the Mental Health Reform to improve the quality, availability, and accessibility of mental health services in Israel [22–24]. Mental health care responsibilities were added to the basket of services provided by all four health maintenance organizations in Israel. Secondly, the COVID-19 pandemic led to a rise in the incidence of mental health disorders [25]. The World Health Organization (WHO) reported a 25% increase in anxiety and depression cases worldwide due to the pandemic [26]. Finally, a war (the Iron Swords War) broke out in October 2023, exposing significant portions of the population to traumatic war-related events [27, 28].

Primary care physicians frequently manage visits, prescriptions, and treatments for depression, anxiety, and severe mental illness [29]. Researchers suggest that seeking help or medication from a PCP for mental health issues may reduce stigma [30]. Additionally, studies consistently demonstrate that mental health conditions such as depression and anxiety are associated with higher health care expenditures; individuals with mood disorders have more than double the annual healthcare expenses compared to those without, even after adjusting for sociodemographic and health-related factors [31–35].

Therefore, many countries incorporate mental health training into their family medicine curricula [36]. These programs offer either optional or mandatory rotations, with durations and settings varying by country—for example, Australia and the UK offer optional rotations, the US has full mandatory rotations, and Brazil provides part-time rotations (5 h weekly for six months) [36]. In Israel, a two-month mental health rotation in an outpatient clinic or hospital is mandatory as part of the family medicine specialty [37].

The purpose of the study was to measure patient visit rates with depression and anxiety to PCPs over time, characterize these patients, and describe their healthcare service consumption within the primary care services, secondary care, emergency room visits, and internal medicine hospitalizations. Assessing service utilization among mental health patients and identifying areas where the system is stressed is crucial for policymakers to advance better planning of integrated care within healthcare systems.

## Methods

### Data source and setting

Maccabi Healthcare Services (MHS) is the second-largest HMO in Israel, providing healthcare services to over a quarter (2.7 million) of Israel’s population. This observational study is based on data extracted from the Maccabi HealthCare database. The database includes demographic data for all members as well as visit and diagnosis records for all outpatient and community-based physician visits, hospitalizations (including diagnoses), and all prescription purchases. MHS has established registries for illnesses, such as hypertension (HTN), cardiovascular disease, and diabetes. Other registries such as a polypharmacy registry (patients purchasing eight or more different medications), a ‘serious illness’ registry (which includes patients undergoing dialysis, diagnosed with Gaucher disease, hemophilia, AIDS, or currently being treated for cancer), and a severe mental illness (SMI) registry for patients with schizophrenia, schizoaffective disorder, and bipolar disorder, were also established [38]. Personal identity numbers allow for the linkage of all recorded data at the individual level, including connections to MHS registries, demographic information, and records of all health-related interactions. However, we acknowledge that some patients may seek care from private physicians or purchase medications outside the MHS system, which are not captured in the data. Therefore, this study includes only patients and transactions documented within MHS.

### Study population

Study participants included patients aged eighteen and older who visited a PCP between 2013 and 2024. The population was divided into three distinct groups for each study year: a depression/anxiety group, the SMI group, and a comparison group. The ‘depression/anxiety’ is the research group, and it includes patients with a diagnosis of depression or anxiety (recorded during any physician visit or hospitalization) or who purchased medication for the treatment of depression or anxiety (purchase of at least one antidepressant or anxiolytic in the year). A broad range of diagnoses indicating depression and anxiety were included: depression, anxiety, post-traumatic stress disorder (PTSD), obsessive–compulsive disorder (OCD), personality disorder, sleep disorder, dual diagnosis, and addiction disorder. The second group comprised all patients in the SMI registry (for patients with schizophrenia, schizoaffective disorder, and bipolar disorder). The third group, the comparison group, included all other patients who had visited a PCP in the year. Each member is categorized annually and may change groups from one year to the next, except for those in the SMI group, which is considered a chronic condition and therefore defined by lifetime presence in the registry. The three groups were mutually exclusive each year.

### Measurements

For each group, demographic and health characteristics were extracted from the database. Socioeconomic status and population group (Arab/Orthodox Jew/Other—general Jewish population and other non-Jewish minorities) were based on the patient’s address and categorized according to census and statistical data reported by the Israel Central Bureau of Statistics. Body mass index (BMI) is measured periodically by physicians, nurses, and dietitians. Patients with a BMI result of 30kg/m^2^ and above were defined as obese. Most recent smoking status (routinely collected during primary care physician visits) was also extracted from the database. Missing values account for less than one percent of cases. However, we are aware that recent documentation is lacking and therefore use an ‘ever smoked’ variable rather than current smoking status. Missing values for obesity and smoking status were imputed as indicating the absence of the respective condition or behavior. Approximately 75% of adults had a recorded BMI measurement within the past three years. We are aware that there is a bias in the documentation of data in medical records, wherein overweight or obese patients are more likely to be noted. It is possible that patients presenting with mental health issues may more likely to be assessed. However, given that obesity was just one of several chronic illnesses included in a composite chronic illness measure, the potential for major bias between the two groups was felt to be minimal.

The number of chronic illnesses was determined based on enrollment in the following MHS registries: hypertension (HTN), heart disease, and diabetes, as well as the presence of obesity and current smoking status**.**

The proportion of patients from each group visiting a PCP was calculated for each year, as well as the proportion of visits from each group in relation to the total primary care visits for that year.

To examine service utilization as a proxy indicator of the total severity of burden, we used the groups mentioned above for the last year of the study (2024). During this period, we calculated the following proportions for each group: patients with at least one emergency room (ER) visit (without hospitalization), patients hospitalized in an internal medicine/acute geriatric unit, patients with at least one visit to a secondary care physician (excluding gynecologists and psychiatrists) and patients who entered the polypharmacy registry. Mean frequencies were calculated for each study year for each measure of interest. Demographic characteristics for the depression/anxiety group were not found to change over time, and therefore, the demographic characteristics of the last study year are presented.

### Statistical analyses

Descriptive statistics were used to summarize the demographic and health characteristics of the study population groups, as well as to present rates and proportions of patients and visits across the study years. Analysis of Variance (ANOVA) was used to compare the mean number of visits to the PCP by group, controlling for age group, gender, population group, socioeconomic status, and number of chronic illnesses.

Logistic regression analysis was used to evaluate the odds ratio of the study groups for various service utilization outcomes, including emergency room visits not resulting in hospitalization, hospitalizations, visits to secondary care physicians, and polypharmacy. To account for differences in patient characteristics between study groups, adjustments were made for age groups (18–34, 35–64, 65 +), gender, socioeconomic status, population group, and the number of chronic illnesses (0, 1, 2 +).

Analysis was performed with The R Project for Statistical Computing, version 4.2.1, and the packages "Tidyverse", "Broom", and "Emmeans", as well as SPSS, version 29 (IBM©).

The study was approved by the MHS Institutional Review Board (IRB, Helsinki Committee approval No. 0008–24-MHS), in accordance with the Declaration of Helsinki. The committee also granted a waiver of informed consent for this study.

## Results

Demographic and health characteristics differed between the three groups (Table 1). Those patients in the depression/anxiety group were more likely to be female, older, and suffer from chronic illness. Orthodox Jews, Arabs, and patients from a lower socioeconomic bracket were under-represented in the depression/anxiety group. In contrast, patients in the SMI group were more likely to be male and younger, with a greater likelihood of coming from a lower socioeconomic bracket.

**Table 1:** Demographic & health characteristics (%) of study population by group. Maccabi HealthCare Services, Israel, January-December 2024

Characteristic	Depression/ anxiety	Severe mental illness	Comparison group
N (%)	283,623 (16.75)	27,439 (1.62)	1,381,643 (81.62)
Gender (% male)	36	53	48
Age group (%)			
18–24	7	6	12
25–34	15	18	20
35–44	15	20	17
45–54	18	22	18
55–64	16	17	15
65–74	14	12	11
75 +	15	6	7
Socioeconomic status (%)			
Low	16	28	20
Medium	46	47	44
High	38	25	36
Population group (%)			
General Jewish pop/other	91	84	86
Orthodox Jew	6	12	8
Arab	3	4	7
% in registry			
Hypertension	31	24	19
Heart disease	14	10	9
Diabetes	15	17	9
Severe disease	4	2	2
Obesity	27	36	21
Smoking (ever)	42	52	32

**Fig. 1:**
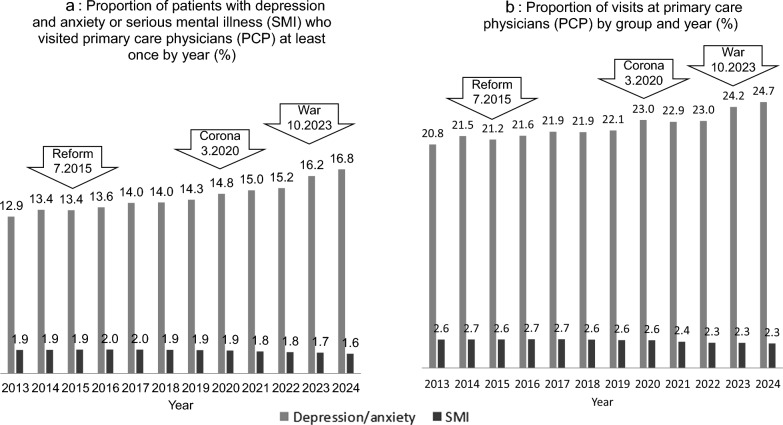
the proportion of patients (**a**) and the proportion of visits (**b**), in 2013–2024 (in %), among patients with depression and anxiety disorders (gray) or serious mental illness (SMI, black). The implementation of the mental health reform (reform). The COVID-19 pandemic (corona) and the Iron Swords war (war) and their respective dates, are noted with arrows

The proportion of patients with depression/anxiety increased annually between 2013 and 2024 (Fig. 1 a), as did the proportion of visits (Fig. 1 b). The annual increase for both measures was incremental, with a more pronounced increase observed, particularly for visits, during the COVID-19 pandemic and during the war.

**Fig. 2:**
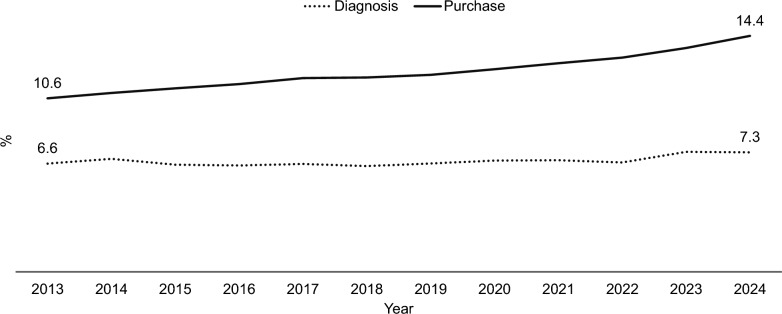
Rates of depression/anxiety by data source (in %) and year. Diagnosis (gray – dashed) and medication purchase (black)

Most of the increase in the depression/anxiety group was attributable to the rise in antidepressant/anxiolytic medication purchase rather than an increase in the number of patients with a diagnosis of depression/anxiety (Fig. 2). Medication purchases increased from 10.5% of the study population in 2013 to 14.4% in 2024, while the proportion identified by diagnosis remained consistent over the decade (7.0%). There is an overlap between them: five percent of the study population had a diagnosis and purchased a prescription.

**Fig. 3:**
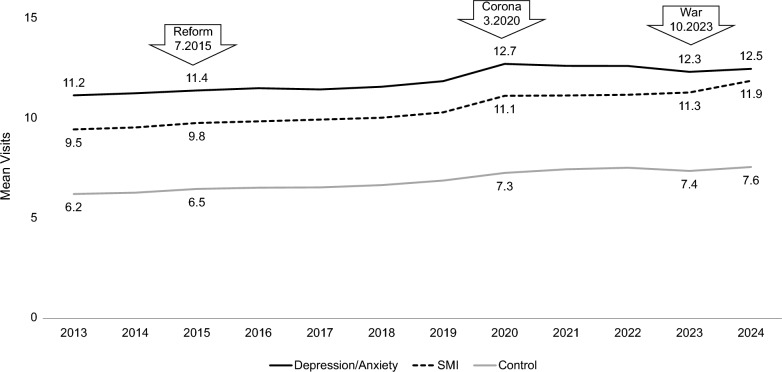
Mean number of visits to the primary care physician (PCP) by group and year. Depression/Anxiety – black line, Severe Mental Illness (SMI) – black dashed line, and control group – gray line. The implementation of the mental health reform (reform), the COVID-19 pandemic (corona), and the Iron Swords war (war) and their respective dates, are noted with arrows. The mean number of visits around each event is marked on the graph

The average number of PCP visits increased over time for all three groups (Fig. 3), with a marked increase at the outbreak of COVID-19. An increase in the average number of visits was also observed mostly among the SMI population during the Iron Swords war. The adjusted mean number of visits in 2024 was 14.2 (95% CI: 14.1–14.4) for the depression/anxiety group, 14.0 (95% CI: 13.7–14.2) for the SMI group, compared to 9.9 (95% CI: 9.8–10.0) for the comparison group.

During 2024, 20.5% of patients with depression/anxiety presented to the ER, and 23.1% of the SMI group, compared to 13.7% of the comparison group (*p* < 0.05). Adjusted odds ratios (OR) indicated that patients with depression/anxiety were 56% more likely to present to the ER than the comparison group (Table 2). Patients in the SMI group were 87% more likely to present to the ER than the comparison group.

**Table 2:** Factors associated with selected service utilization (logistic regression model). Maccabi HealthCare Services, Israel, 2024

Characteristic	Category	N	Emergency room		Hospitalization		Secondary care specialist		Polypharmacy	
			OR	95% CI	OR	95% CI	OR	95% CI	OR	95% CI
Group	Comparison	1,378,965								
	Depression/Anxiety	283,118	1.6	1.5–1.6	2.0	2.0–2.1	1.4	1.4–1.4	2.4	2.3–2.5
	Severe mental illness	27,403	1.9	1.8–1.9	2.9	2.7–3.0	0.9	0.8–0.9	2.5	2.2–2.8
Age group	18–34	511,144								
	35–64	843,366	0.7	0.7–0.7	1.5	1.4–1.6	1.2	1.2–1.1	3.6	3.1–4.2
	65 +	334,976	0.7	0.7–0.7	3.2	3.1–3.4	1.5	1.4–1.5	4.5	3.9–5.3
Gender	Female	907,660								
	Male	781,826	0.8	0.8–0.8	1.3	1.3–1.3	0.4	0.4–0.4	1.4	1.3–1.5
Socioeconomic status	Low	328,492	1.3	1.3–1.3	1.6	1.5–1.6	0.6	0.6–0.7	1.2	1.2–1.3
	Medium	751,010	1.2	1.2–1.2	1.3	1.3–1.3	0.8	0.8–0.8	1.1	1.1–1.2
	High	609,984								
Population group	General Jewish population/Other	1,464,130								
	Orthodox Jew	124,593	1.0	1.0–1.0	0.9	0.9–1.0	0.9	0.9–0.9	0.9	0.8–1.0
	Arabs	100,763	1.3	1.3–1.3	1.2	1.2–1.3	0.8	0.8–0.8	1.3	1.2–1.4
Number of Chronic illnesses	0	1,163,728								
	1	290,707	1.2	1.2–1.2	2.6	2.5–2.7	1.5	1.5–1.5	6.4	5.9–6.9
	2 +	235,051	1.8	1.8–1.8	7.3	7.0–7.5	2.7	2.6–2.7	12.3	11.3–13.3

Internal medicine hospitalization rates were 5.0% among depression/anxiety patients and 5.6% among patients in the SMI group, compared to 1.8% in the comparison group (*p* < 0.05). After adjustment, both the depression/anxiety group and the SMI group were more than twice as likely to be hospitalized at least once than the comparison group (Table 2).

Of all patients with depression/anxiety, 84.2% visited a secondary care physician at least once, compared to 75.3% in the comparison group and 72.3% in the SMI group (*p* < 0.05). After adjustment, depression/anxiety patients were 38% more likely to visit a secondary care physician than the comparison group. The SMI group was 13% less likely than the comparison group to present to a secondary care physician (Table 2).

Of patients with anxiety/depression, 1.1% were registered in the polypharmacy database compared to 1.0% in the SMI group and 0.3% in the comparison group. After adjustment, both the depression/anxiety and SMI groups were twice as likely to be in the polypharmacy registry than the comparison group (Table 2).

## Discussion

This study examined the prevalence, sociodemographic characteristics, and healthcare utilization of patients diagnosed with depression and anxiety in Israel over the past 12 years, starting from 2013.

From 2013 to 2024, there was a significant increase in the number of patients with depression and anxiety visiting PCPs. This trend accelerated in recent years, particularly during the COVID-19 pandemic and the Iron Swords war. Additionally, individuals with severe mental illness (SMI) demonstrated higher average visit rates during the war.

The ‘depression/anxiety’ group was mainly female (64%), older, and from higher socioeconomic backgrounds compared to the SMI and control groups. This is consistent with previous studies showing increased mental health diagnoses among older women [39–41]. Minority groups, such as Ultra-orthodox Jews and Arabs, had lower rates of mental health diagnoses, compared to their representation in the MHS population and despite similar health care services. Their lower rates in the study may be due to stigma and cultural factors discouraging care-seeking [40, 42]. However, these low rates may be genuine, as research indicates that protective factors such as robust social support systems and engagement in religious beliefs and practices may significantly contribute to reduced rates of depression and anxiety [43–48].

Patients with depression or anxiety visited the PCP more frequently, had higher rates of emergency room visits (OR 1.56) and secondary care physician visits (OR 1.38), hospitalization in internal medicine departments (OR 2.03), and higher rates of polypharmacy (OR 2.38). Our findings underscore the increasing economic burden patients with depression/anxiety place on the healthcare system, compounded by their older age and higher prevalence of chronic diseases. This aligns with studies indicating that the combination of depression or anxiety with other comorbidities exacerbates health outcomes [3–11, 49, 50].

Yet, after adjustments for age and chronic morbidity, the high utilization rates amongst those with depression/anxiety remained consistent, highlighting the need for boosting treatment resources for patients with depression and anxiety. Emphasis should be placed on holistic and comprehensive care provided by PCPs who are trained in both physical and mental health. This aligns with the rationale of the Mental Health Reform, which aimed to combine mental and chronic physical treatments, removing the barriers and stigma, and transition to integrated care within community settings [22–24].

SMI patients had fewer secondary care visits than comparison patients (OR 0.87). This finding aligns with evidence of underuse, especially for physical health, despite more emergency admissions and comorbidities. Factors like low socioeconomic status, minority ethnicity, rural location, and social isolation are linked to reduced outpatient and secondary care access for SMI patients, leading to healthcare disparities [51–54].

Researchers recommended expanding community access to both pharmacological and psychological mental health interventions [12, 14, 21]. Evidence supports a community-based model, re-shaping PCPs’ role to manage mental health disorders and reduce psychiatrist wait times [24, 55–57].

The rise in demand for healthcare resources by patients with depression and anxiety necessitates significant conceptual and systemic changes. We suggest three major policy changes to address this growing need: the addition of trained mental health professionals to provide treatment and integrative follow-up in the community, increasing primary care physicians’ (PCPs) training time regarding mental health issues, and revising the incentive and payment model for PCPs to ensure it promotes quality and effective care through specialized training.

First, the addition of trained mental health professionals in the community is crucial.

Community-based psychological interventions have improved mental and physical health, enhanced patient well-being for those with depression and anxiety, and lessened the burden on primary care services [12]. Thus, we suggest matching patients to appropriate health workers based on symptom severity after professional triage. Mild cases that do not need medication can be treated by trained psychologists, social workers, or mental health students. This approach enables prompt, tailored care and eases pressure on the mental health system [58–60].

Second, the study showed an increase in visits to PCPs in all groups, especially in the SMI group. Therefore, we believe that enhancing PCPs’ mental health training is needed. Comprehensive programs help PCPs manage depression and anxiety more effectively, improving patient outcomes and reducing service use [21]. Additionally, collaborative care models with extended PCP training yield significantly better results [16, 18].

Extending the duration of psychiatry training for family medicine residents from 2 months to at least 6 months may be beneficial [36]. Also, focusing rotations on community mental health clinics, instead of psychiatric hospitals, would provide more relevant experience with depression, anxiety, and chronic comorbidities. With improved training, family physicians could manage these cases, while psychiatrists would address more severe mental illnesses [61].

Finally, revising the incentive and payment model for primary care providers (PCPs) may facilitate the delivery of high-quality care. Current models often do not adequately compensate PCPs for managing patients with complex mental health needs. Evidence indicates that incentive models for family physicians can produce modest improvements in the management of depression and anxiety within primary care settings. Financial and organizational incentives have been linked to slight increases in counseling, psychotherapy, and antidepressant prescribing rates [62–64]. Structural barriers, including certain payment systems and organizational arrangements that do not facilitate evidence-based practice, may limit optimal care delivery. While some research suggests that aligning incentives with evidence-based practices could enhance care quality and sustainability, findings also indicate that incentives alone are insufficient [65]. Improvements in managing depression and anxiety typically require multifaceted approaches, such as collaborative care models and systematic follow-up, integrated with wider organizational changes [66]. Further research is necessary to support these policy recommendations.

In summary, the need for mental health professionals has outstripped availability and will continue to rise following the Iron Swords war. Therefore, systemic changes are needed. We recommend introducing alternative mental health specialists trained to manage depression and anxiety alongside psychiatrists and psychologists, improving access to care. PCP’s training should focus more on mental health, preparing physicians to serve this group effectively. Policymakers should extend family medicine mental health training, shift some training from hospitals to community clinics, and update reimbursement models for PCPs to better meet the growing need for mental health services.

This study has several limitations. First, it is an observational/descriptive analysis rather than a prospective or longitudinal one, so causality cannot be established. The observed increase in visits during the war or COVID period cannot be conclusively attributed to these events. Other contributing factors, such as changes in coding practices, potential misclassifications of mental health diagnosis, provider incentives, or mental health campaigns, may have influenced the findings. The actual rates of anxiety and depression may be higher than those reported. Under-reporting may be attributed to concerns about stigma and the documentation of a mental health diagnosis in medical records. Additionally, some patients may feel uncomfortable discussing mental health issues with their primary care physician, resulting in complaints that are more somatic in nature. Consequently, diagnoses such as musculoskeletal pain, abdominal discomfort, or shortness of breath may reflect psychosomatic conditions that are not necessarily recorded as mental health conditions. Conversely, the reported increase might be exaggerated, secondary to greater awareness and readiness for treatment rather than an actual rise in cases. Diagnoses and medication purchases during the study period could have increased because of external factors, such as media advertisements following the mental health reform that encouraged patients to consult their PCP. This likely enhanced the representation of the depression/anxiety group but not the SMI group, as shown in Fig. 1. Nonetheless, this can be seen as a positive outcome of the health reform, which aims to break the mental-physical barrier.

Additional limitation of this study is the absence of direct measures of mental illness severity and physical health status. Due to the nature of the administrative data used, detailed clinical assessments and standardized diagnostic scales were not available. This restricts our ability to account for important confounding factors that may influence healthcare utilization, such as underlying health conditions or clinical complexity. Although we attempted to partially adjust for health status using registry-based indicators, these proxies may not fully capture the nuances of patient health. Future research incorporating richer clinical data and validated severity measures is needed to better understand the drivers of increased service use among individuals with common mental disorders.

This study used computerized data from MHS medical records. Some data, such as obesity and smoking status, were missing, which could bias estimates. However, the missing data regarding ‘smoking’ was limited to less than one percent of the study population. As obesity was only one of the chronic illness conditions measured, major bias is unlikely.

We also acknowledge that some patients may seek care from private physicians or purchase medications outside the MHS system, which are not captured in the data. Finally, orthodox Jews, Arabs, and patients with low socioeconomic status exhibited low rates of depression and anxiety. This may stem from fear of stigmatization or tagging, as well as underrepresentation, underdiagnosis or measurements bias. Further studies should investigate these populations.

## Conclusion

Patients with depression and anxiety are primarily female, older, of lower socioeconomic status, and have chronic illness. From 2013 to 2024, the number of such patients increased, especially during the COVID-19 pandemic and the Iron Swords war. These patients utilized medical services more frequently than the general population, averaging 14.2 visits per year compared to 9.8, including more emergency room and consultant visits, hospitalizations, and medications.

The rise in demand for healthcare resources by this group necessitates conceptual and systemic change by three major policy changes: the addition of trained mental health professionals to provide treatment and integrative follow-up in the community, increasing PCPs’ training time regarding mental health issues, and revising the incentive and payment model for PCPs to ensure it promotes quality and effective care through specialized training.

Emphasizing comprehensive care provided by primary care physicians trained in physical and mental health and enhancing primary care physician training programs in mental health, will better prepare physicians to treat these populations, and according to our study may potentially improve health outcomes, and reduce service usage and costs.

## Supplementary Information

Additional file 1.

## Data Availability

The datasets generated and/or analyzed during the current study are not publicly available due to IRB restrictions but are available from the corresponding author on reasonable request.

## Abbreviations

BMI: Body mass index
ER: Emergency room
HTN: Hypertension
MHS: Maccabi healthcare services
OCD: Obsessive compulsive disorder
PCP: Primary care physician
PTSD: Post traumatic stress disorder
SMI: Serious mental illness
WHO: World health organization

## Declarations

### Transparency declaration

I, Marina Mor Shalon, affirm that the manuscript is an honest, accurate, and transparent account of the study being reported; that no important aspects of the study have been omitted; and that any discrepancies from the study as planned (and, if relevant, registered) have been explained.
